# Early COVID-Related pandemic impacts and subsequent opioid outcomes among persons receiving medication for opioid use disorder: a secondary data analysis of a Type-3 hybrid trial

**DOI:** 10.1186/s13722-023-00409-7

**Published:** 2023-09-13

**Authors:** Tim Janssen, Bryan R. Garner, Julia Yermash, Kimberly R. Yap, Sara J. Becker

**Affiliations:** 1 Department of Behavioral and Social Sciences, Brown University School of Public Health, 121 South Main Street, Providence, Rhode Island 02903 USA; 2 Department of Internal Medicine, Ohio State University College of Medicine, 2050 Kenny Road, Columbus, OH 43221 USA; 3 Center for Dissemination and Implementation Science, Northwestern University, 633 North St Clair, Chicago, IL 60611 USA

**Keywords:** COVID-19, Opioids, Epidemic-pandemic impacts inventory, Opioid use disorder

## Abstract

**Background:**

Opioid overdoses have continued to increase since the start of the COVID-19 pandemic. The pathways through which the COVID-19 pandemic has affected trajectories of opioid use and opioid-related problems are largely unknown. Using the Epidemic-Pandemic Impacts Inventory (EPII), a novel instrument that assess pandemic-related impacts across multiple life domains, we tested the hypothesis that COVID-related impacts (on e.g., interpersonal conflict, employment, infection exposure, and emotional health) experienced in the early months of the pandemic would predict changes in opioid use and opioid-related problems at follow-up.

**Methods:**

This analysis was embedded within a cluster randomized type 3 implementation-effectiveness hybrid trial that had enrolled 188 patients across eight opioid treatments prior to the start of the pandemic. Participants had all been recently inducted on medication for opioid use disorder and were actively receiving treatment. Participants reported on their opioid use and opioid-related problems at baseline and 3-, 6-, and 9-month post-baseline assessments. Between May and August 2020, participants were sent an optional invitation to complete the EPII.

**Results:**

One hundred thirty-three respondents completed the EPII and 129 had sufficient data to analyze the EPII and at least one subsequent follow-up. In logistic and zero-inflated negative binomial analyses adjusting for covariates, each endorsed pandemic impact in the interpersonal conflict domain was associated with 67% increased odds of endorsement of any opioid use, and each impact in the employment and infection exposure-domains was associated with 25% and 75% increases in number of endorsed opioid-related problems, respectively.

**Conclusions:**

Mitigating the effect of the pandemic on patients’ interpersonal relationships and employment, and promoting greater infection control in opioid treatment programs, could be protective against negative opioid-related outcomes.

*Trial registration* The present study describes secondary data analysis on a previously registered clinical trial: clinicaltrials.gov/ct2/show/NCT03931174.

**Supplementary Information:**

The online version contains supplementary material available at 10.1186/s13722-023-00409-7.

## Background

Prior to the COVID-19 pandemic, the opioid epidemic was deemed a public health emergency in the United States [1], causing more deaths per year than car accidents and violent crime [2]. Since the onset of the pandemic, overdoses and opioid-related sequalae have continued to increase. In the 12-month period ending April 2021, fatal opioid-related overdoses increased 35% relative to the year before and the number of lives lost due to opioid overdose surpassed 100,000 for the first time in United States history [3].

The specific pathways through which COVID-19 has exacerbated opioid use and opioid-related problems are not well understood. One potential pathway was through social distancing that, while essential to flattening the curve, placed major constraints on employment, social activities, and childcare –protective factors against opioid misuse [4, 5]. Moreover, the social isolation created by COVID-19 has been associated with increased prevalence and severity of substance use, mental health, and physical health problems [6, 7], all of which have been linked to increased opioid misuse [8, 9]. Persons with opioid misuse are also at heightened risk of exposure to COVID-19 due to both direct (i.e., effects of opioid use on respiratory health) and indirect (i.e., restricted access to healthcare) pathways [10]. Perhaps most concerningly, reports of interpersonal conflict and violence increased in the early months of the pandemic [11, 12]. Interpersonal conflict is a major risk factor associated with opioid use disorders (OUDs), prescription drug use, heroin use, and opioid overdose [13, 14].

Systematically examining the impact of pandemic-related experiences on patient functioning has been difficult due the lack of assessment tools. The Epidemic-Pandemic Impacts Inventory (EPII) is a new measure developed by Grasso and colleagues [15] as a comprehensive assessment tool to identify potentially modifiable risk factors caused by the pandemic that are associated with increases in physical and mental health issues, such as increases in substance use and social isolation. A key feature of the EPII is its examination of these COVID-19 pandemic-induced risk factors across multiple life domains including employment, social isolation, infection exposure, emotional health, and interpersonal conflict.

In the current study, we assessed the longitudinal associations between COVID-19 experiences during the early months of the pandemic, and subsequent opioid use and opioid-related problems among persons receiving medication to treat OUD. We sought to investigate which specific EPII domains were most strongly associated with opioid-related outcomes. Due to the paucity of prior data, our analyses of specific EPII domains were exploratory, though we conjectured that interpersonal conflict-related impacts would have the largest effect on subsequent opioid use and opioid-related problems.

## Methods

### Parent study

Data collection was embedded within a cluster-randomized type 3 implementation-effectiveness hybrid trial called Project MIMIC (Maximizing the Implementation of Motivational Incentives in Clinic, clinicaltrials.gov/ct2/show/NCT03931174). Project MIMIC is focused on evaluating two multi-level implementation strategies for helping Opioid Treatment Programs (OTPs) and their staff implement contingency management, an evidence-based behavioral intervention in which patients earn prizes for meeting treatment goals [16]. The control condition was the Addiction Technology Transfer Center (ATTC) Network implementation strategy, featuring three components: didactic training, performance feedback, and ongoing consultation. The experimental condition was an enhanced ATTC (E- ATTC) strategy, with an added Pay-for-Performance extrinsic component and Implementation Sustainment Facilitation as an intrinsic component.

To qualify for Project MIMIC, participants had to be at least 18 years old and have been inducted on medication for OUD within the last 30 days. Participants also had to be in active treatment at one of the partner OTPs, which typically consisted of daily medication dispensing, weekly group and/or individual counseling sessions, and periodic case management sessions [17]. All participants completed a baseline survey upon enrollment (see Measures). In addition, participants were invited to complete standard follow-up assessments at interviews occurring 3-, 6-, and 9-months post-baseline. All data collection was performed in accordance with IRB-approved procedures.

When social distancing regulations began in March of 2020, Project MIMIC had enrolled and obtained informed consent from 188 patients receiving treatment at eight OTPs across the New England region. Among this sample, methadone was the most commonly received medication (88%), followed by buprenorphine (11%), and naltrexone (1%). In the early months of the COVID pandemic, all 188 participants in this first cohort of the parent clinical trial were invited to provide informed consent for a supplementary survey on COVID-19-related impacts using the EPII assessment tool.

### Assessment procedures

The EPII assessment was administered between May-August 2020, an average of 5.77 months (*SD* = 1.94) after cohort baseline. After providing informed consent for this supplemental data collection, participants completed the EPII and received a $20 gift card.

The opioid-related outcomes for this analysis were selected from the first subsequent assessment following EPII assessment. These assessments were the standard follow-up interviews for Project MIMIC. For most participants, the first assessment subsequent to the EPII assessment was at the 9-month mark (86.9%). For the remainder of participants, the first assessment subsequent to the EPII assessment was at the 6-month mark. Among those who completed the EPII, 97% and 87% completed the 6- and 9-month assessments, respectively.

### Measures

#### Demographics

Upon enrollment, participants answered questions about their sociodemographic characteristics. Focal covariates included sex assigned at birth (male/female), a dichotomous variable coded to represent racial/ethnic identity (Non-Hispanic White vs. racially/ethnically minoritized people), and age in years, since these variables have demonstrated associations with opioid use in prior studies [18]. Expanded sociodemographic descriptors of the sample are included in the Results section.

#### Opioid-related outcomes

At baseline and each follow-up assessment, participants completed the Timeline Follow-back Interview [19], which assessed days of use of heroin and other opioid use (excluding medications like methadone and buprenorphine taken as prescribed) over the past 30 days. Participants also completed a brief measure of opioid-related problems over the past 30 days, an 11-item scale adapted from the well-validated Global Appraisal of Individual Needs Substance Problem Scale [20, 21] to focus on opioids. The measure of opioid-related problems had high internal consistency (α = 0.95), consistent with published psychometric data for the scale [21].

#### EPII Survey

The EPII [15] consists of 92 binary (yes/no) questions designed to inventory ways the COVID-19 pandemic may have affected respondents. We added five questions about substance-related impacts: three items about access to harm reduction services (access to naloxone, sterile injection equipment, and recovery support), one item about access to their preferred substance, and one item about access to take-home doses of medication for OUD. Next, we computed domains as in Grasso and colleagues [15], but reduced some of the large domains into smaller subdomains to facilitate specific research interest: (a) six items in the home life domain were shifted into a new domain on interpersonal conflict; (b) four items in the home life domain and two items in the emotional health domain were shifted into a new domain on caretaking; and (c) two items in the employment domain about housing instability were shifted into the economic domain, which assesses the ability to afford basic needs (e.g., food, water, rent). Items related to positive impacts of the pandemic and impacts of the pandemic on others in the home or in the workplace were excluded (see Additional file 1: Table S1). The final analysis contained 70 EPII items across the following 10 domains: employment, interpersonal conflict, social, economic, emotional health, substance use, physical health, quarantining and physical distancing, infection exposure, and caretaking (αs = 0.58 − 0.83).

### Statistical analysis

The analytical sample was defined as EPII survey completers with at least one follow-up assessment. Preliminary analyses examined whether condition (assignment to implementation strategy condition), or contingency management dosage (number of sessions received) were associated with the focal items but no significant associations were identified. Similarly, exploratory analyses demonstrated that neither assessment origin (outcome assessed at 6- or 9-month mark) nor differences in the lag between the EPII assessment and the next subsequent assessment predicted opioid-related outcomes. Thus, data were pooled across conditions (with condition effects explicitly examined in adjusted models), and none of the other study design features (number of sessions, assessment origin, assessment lag) were controlled for in analyses.

We examined associations between EPII domains and opioid use/problems in two steps. First, we examined Pearson correlations between EPII domains and baseline number of days of opioid use, as well as baseline number of opioid-related problems. Second, we estimated multivariate longitudinal models to obtain adjusted associations between EPII domains, featuring associations between other EPII domains, baseline opioid use/problems, condition assignment, age, sex assigned at birth, and racially/ethnically minoritized status as covariates. Follow-up opioid use was modeled as a binomial (yes/no: any opioid use days in past 30 days at follow-up) outcome because 75% of participants did not endorse any past-30-day opioid use, leading to scant data for count analysis. Participants endorsed an average of 2.0 out of 11 problems (SD: 3.5), with 62.4% endorsing zero problems. As such. follow-up opioid-related problems was analyzed with a zero-inflated negative binomial distribution, predicting: (1) the probability of being an excess zero (outside the expected distribution), and (2) the count value, if not zero (see Atkins and colleagues, [22]. Our focal outcome was the count distribution, i.e. the effect of COVID-related impacts on predicting number of opioid-related problems.

## Results

### Sample characteristics

A 71% response rate was obtained for the EPII (*n* = 133) and respondents were representative of the full sample in terms of racial and ethnic identity, household income (where available, *n* = 152 out of *N* = 188), and age, but non-completers more often identified as male (X^2^(1, *N* = 186) = 2.790, *p* = .006). Among the 133 participants that completed the EPII, 83% (*n* = 110) reported being Non-Hispanic White; 4.5% (*n* = 6) reported being Black or African American, 4.5% (*n* = 6) reported Other race, 3.8% (*n* = 5) reported being of more than one race, 1.5% (*n* = 2) reported being Hawaiian or Pacific Islander, 1.5% (*n* = 2) reported being American Indian or Alaska Native, and 0.8% (*n* = 1) reported being Asian. 10% (*n* = 13) of participants reported being of Hispanic or Latin descent and 60% (*n* = 80) of the sample indicated female sex at birth. Patients reported a mean age of 36 years (*SD* = 9.8), and 82% (*n* = 109) of the sample reported having attained high school completion. Based on *n* = 109 patients who completed questions on income, mean household income was $32,980 (*SD* = $40,367). Complete data from the EPII and at least one subsequent follow-up assessment were available from 129 respondents (98% of EPII-completers, 69% of parent-trial participants), which was defined as the final analysis sample.

### Bivariate correlations and regression models

Table 1 shows inter-domain correlations and descriptives for the EPII domains. Inter-domain correlations were significant, suggesting the presence of a shared underlying factor, but the absolute correlation coefficients were not large enough to indicate problematic multicollinearity among the scales (*r*’s < 0.70) [23].

**Table 1:** Pearson correlations among the epidemic-pandemic impacts inventory (EPII), opioid outcomes, and descriptive statistics

Scale name (number of items)	Associations among EPII domains and T2 opioid outcomes											Descriptive statistics
	2	3	4	5	6	7	8	9	10	11	12	M (SD) / %
1. Employment (6)	0.49***	0.39***	0.43***	0.29***	0.29***	0.23**	0.32***	-0.02	0.33***	0.147	0.169	1.47 (1.47)
2. Interpersonal conflict (6)		0.45***	0.47***	0.35***	0.38***	0.21*	0.37***	0.04	0.46***	0.243***	0.156	1.05 (1.27)
3. Social isolation (10)			0.48***	0.52***	0.37***	0.54***	0.42***	0.20*	0.32***	0.134	0.101	5.71 (2.71)
4. Economic (7)				0.48***	0.41***	0.37***	0.42***	0.14	0.22*	0.106	0.249***	1.86 (1.67)
5. Emotional health (5)					0.27**	0.46***	0.37***	0.24**	0.25**	− 0.016	0.090	1.97 (1.43)
6. Substance use (5)						0.35***	0.29***	0.20*	0.20*	0.184**	0.261***	0.31 (0.94)
7. Physical health (7)							0.5***	0.19*	0.17	− 0.013	0.037	3.23 (1.79)
8. Physical distancing (10)								0.4	0.09	0.091	0.143	1.93 (1.89)
9. Infection exposure (7)									–0.09	0.057	0.190**	0.23 (0.49)
10. Caretaking (7)										–0.006	0.014	1.15 (1.89)
11. Any Opioid Days Y/N (1)											0.392***	75%
12. Opioid-Related Problems (11)												2.02 (3.48)

*Note*. *p < .05, **p < .01, ***p < .001.

Table 2 shows the regression results for opioid use and opioid-related problems, respectively. In the logistic regression analysis, higher interpersonal conflict (adjusted OR: 1.65, 95% CI 1.02–2.67) significantly increased the odds of past 30-day opioid use: endorsing an additional COVID-related impact in the interpersonal conflict domain was associated with a 65% increase in odds of past 30-day opioid use.

**Table 2:** Results from multiple regression of opioid outcomes on EPII scales at follow-up (n = 129)

	Any opioid days Y/N			Number of opioid-related problems		
	95% CI			95% CI		
	OR	Lower	Upper	RR	Lower	Upper
Baseline	1.06	1.02	1.09	1.02	0.91	1.13
Employment	1.22	0.81	1.85	**1.25**	**1.08**	**1.43**
Interpersonal conflict	**1.67**	**1.01**	**2.74**	0.95	0.80	1.13
Social isolation	1.09	0.84	1.41	1.02	0.91	1.15
Economic	0.98	0.67	1.43	1.16	0.91	1.48
Emotional health	0.78	0.48	1.26	0.95	0.81	1.12
Substance use	1.36	0.76	2.44	0.95	0.72	1.26
Physical health	0.85	0.58	1.23	1.02	0.85	1.23
Physical distancing	0.92	0.64	1.32	0.86	0.70	1.06
Infection exposure	1.29	0.41	4.07	**1.75**	**1.17**	**2.62**
Caretaking	0.78	0.58	1.06	0.93	0.77	1.12
Age (years)	1.01	0.96	1.07	0.99	0.97	1.01
Condition	1.04	0.39	2.76	0.82	0.50	1.34
Biological sex	1.98	0.66	5.96	1.15	0.58	2.29
Race/ethnicity	1.60	0.47	5.46	1.55	0.88	2.73

*Note*: Condition is coded 0 = control strategy, 1 = experimental strategy; biological sex is coded 0 = male, 1 = female; race/ethnicity is coded 0 = Non-Hispanic White, 1 = racially/ethnically minoritized people. Opioid-related problems regression estimates are results from the count portion of a zero-inflated regression. Results from inflation portion are included in supplementary info (Additional file 1: Table S2). The effect of the addition of all EPII variables to the Any Opioid Days outcome was χ2 (10, N = 128) = 11.7, p = .303. The effect of addition of all EPII variables to the Opioid Problems model was χ2 (10, N = 128) = 14.3, p = .161

In the full zero-inflated model (controlling for all domains simultaneously including baseline opioid-related problems, covariates, and condition assignment), a higher employment domain score (adjusted RR: 1.25, 95% CI 1.04–1.51) and higher infection exposure domain score (adjusted RR: 1.57, 95% CI 1.10–2.24) were associated with an increase in past month opioid-related problems.

## Discussion

This exploratory study identified significant temporal relationships between pandemic-related impacts and subsequent opioid use and opioid-related problems among patients receiving medication for OUD. Specifically, pandemic-related impacts in the interpersonal conflict domain were associated with an increased odds of past 30-day opioid use, but not with likelihood of opioid-related problems. Similarly, employment and infection exposure were both associated with an increased count of opioid-related problems, but not with increased odds of past 30-day opioid use. Although this study was exploratory, we had expected interpersonal conflicts to be one of the strongest predictors of opioid use and opioid-related problems and these results partially confirmed our expectations. The significant effect of pandemic-related interpersonal conflict on subsequent use of opioids is consistent with a wealth of research indicating that interpersonal conflict predicts opioid use and relapse. Our finding that employment and infection control factors predicted subsequent opioid-related problems, and were more important predictors than the other domains of the EPII, is also congruent with literature on risk and protective factors for patients with opioid use disorder. A body of research has documented the central importance of employment as a protective factor for patients in opioid use treatment. and a number of studies in the early months of the COVID-19 pandemic reported a strong association between infection exposure and opioid use. When attempting to conjecture why some EPII domains were associated with one opioid outcome and not the other, it is important to remember that study participants had recently started medication for opioid use disorder and rates of opioid use and opioid-related problems were relatively low across the sample. We therefore believe it is more important to focus on the overall pattern of results–and specifically which of the EPII domains predicted opioid outcomes in the early months of the pandemic–rather than on differences between the two opioid outcomes.

Results of this study highlight potential avenues for policy efforts to mitigate the consequences of pandemic-induced consequences on persons in OUD treatment. Specifically, efforts during future waves of COVID-19 and/or other pandemics could aim to buttress against the harmful co-occurrence of opioid use and interpersonal conflict (e.g., campaigns designed to increase detection of domestic conflict or violence), promote vocational stability (e.g., therapeutic vocational programs at opioid treatment programs), and encourage virus control in OTPs (e.g., rapid testing and free masks widely available to patients at the time of dosing), as a means of protecting against deleterious opioid use outcomes.

Strengths of this analysis included the ability to collect data on early pandemic impacts among a historically marginalized population at high risk of pandemic-related sequalae. Data collection was embedded in an ongoing longitudinal trial [17], which provided real-time data from patients receiving treatment across eight OTPs, increasing the ecological validity of the data. Additionally, use of the EPII enabled a multi-dimensional examination of pandemic-related impacts and their relation to trajectories of opioid use and opioid-related problems.

Study strengths must be interpreted in the context of limitations. First, the EPII survey was voluntary, and while there were no major systematic differences observed between completers and non-completers beyond differences in sex at birth, it remains possible that non-completers were those more likely to experience and/or report negative COVID-19 impacts. Second, given the parent study was an implementation-effectiveness hybrid trial that prioritized measurement of organization- and provider-level data over measurement of patient-level data, it is not possible to conclude whether the changes in opioid use and/or opioid-related problems observed in this study were associated with change in subsequent risk of opioid overdose. Third, impacts on patients’ life domains are likely dynamic. The impacts reported in this study were experienced in the early months of the pandemic in the New England region, yet findings may offer insight into experiences among those recovering from opioid use disorders in the current period of relaxing restrictions and in any future pandemic period. Additionally, the current study did not assess the extent of boredom experienced by the sample as a function of isolation, as this boredom might have represented a closer proxy for decisions to engage in opioid use and/or the extent of experiences of craving. Finally, there were generally very few members of distinct non-White racial and categories (and few participants indicating Hispanic or Latin ethnicity) present in the analytical sample. In the context of logistic regression and zero-inflated count regression, sparse cell coverage could have potentially led to misestimation of effects based on distinct categories of ethnic and racial minoritization. For this reason, a combined dichotomous variable representing non-Hispanic White identity, compared to any other identity, was created. This limited our ability to identify and do justice to crucial differences in pandemic experiences that may have been encountered by members of distinct racial and ethnic groups.

## Conclusions

This timely study provides rare longitudinal data on an evolving issue impacting a high-need patient population. This population was at particular risk during the global COVID-19 pandemic, as the increased market penetration of fentanyl observed during this time means that any instance of opioid use would be more likely to result in overdose [24]. As the effects of the pandemic evolve and threats of future pandemics linger, more data on within-person and between-cohort differences in pandemic impacts and its relation to ongoing opioid outcomes should guide policy efforts to mitigate the negative impacts of pandemics on opioid use and opioid-related problems.

### Supplementary Information

**Additional file 1: Table S1.** Revised domains of the epidemic-pandemic impact inventory (EPII). **Table S2.** Results from inflation component of zero-inflated binomial prediction of follow-up opioid-related problems (n = 129).

## Data Availability

Data and analysis syntax will be made available upon reasonable request once data collection for the primary study is completed (May 2023; collection of measures for the current investigation is already completed).

## Abbreviations

COVID-19: Coronavirus disease of 2019
EPII: Epidemic-pandemic impacts inventory
MIMIC: Maximizing the implementation of motivational incentives in clinic
ATTC: Addiction technology transfer center (also:study control condition)
E-ATTC: Enhanced addiction technology transfer center (also:study experimental condition)
OTP: Opioid treatment program
OUD: Opioid use disorder
IRB: Institutional review board
OR: Odds ratio
RR: Rate ratio
