# Supplementary material for: Early COVID-Related pandemic impacts and subsequent opioid outcomes among persons receiving medication for opioid use disorder: a secondary data analysis of a Type-3 hybrid trial
Source: Addict Sci Clin Pract. 2023 Sep 13;18:54. doi: 10.1186/s13722-023-00409-7 (PMC10500804; doi:10.1186/s13722-023-00409-7)
Supplement: Supplementary file 1 — Additional file 1: Table S1. Revised domains of the epidemic-pandemic impact inventory (EPII). Table S2. Results from inflation component of zero-inflated binomial prediction of follow-up opioid-related problems (n = 129). [file 13722_2023_409_MOESM1_ESM.docx]

SUPPLEMENTAL FILES

Additional Table S1

*Revised domains of the Epidemic-Pandemic Impact Inventory (EPII)*

| **Revised EPII domain** | **Item number** | **Item** | **Initial EPII domain** |
| --- | --- | --- | --- |
| Employment | 1 | Laid off from job or had to close own business. | Work and employment |
|  | 2 | Reduced work hours or furloughed. | Work and employment |
|  | 3 | Had to lay-off or furlough employees or people supervised. | Work and employment |
|  | 6 | Increase in workload or work responsibilities. | Work and employment |
|  | 7 | Hard time doing job well because of needing to take care of people in the home. | Work and employment |
|  | 8 | Hard time making the transition to working from home. | Work and employment |
| Interpersonal conflict | 16 | More conflict with child or harsher in disciplining child or children. | Home life |
|  | 22 | Increase in verbal arguments or conflict with a partner or spouse. | Home life |
|  | 23 | Increase in physical conflict with a partner or spouse. | Home life |
|  | 24 | Increase in verbal arguments or conflict with other adult(s) in home. | Home life |
|  | 25 | Increase in physical conflict with other adult(s) in home. | Home life |
|  | 26 | Increase in physical conflict among children in home. | Home life |
| Social isolation | 27 | Separated from family or close friends. | Social activities |
|  | 28 | Did not have the ability or resources to talk to family or friends while separated. | Social activities |
|  | 29 | Unable to visit loved one in a care facility (e.g., nursing home, group home). | Social activities |
|  | 30 | Family celebrations cancelled or restricted. | Social activities |
|  | 31 | Planned travel or vacations cancelled. | Social activities |
|  | 32 | Religious or spiritual activities cancelled or restricted. | Social activities |
|  | 33 | Unable to be with a close family member in critical condition. | Social activities |
|  | 34 | Unable to attend in-person funeral or religious services for a family member or friend who died. | Social activities |
|  | 35 | Unable to participate in social clubs, sports teams, or usual volunteer activities. | Social activities |
|  | 36 | Unable to do enjoyable activities or hobbies. | Social activities |
| Economic | 20 | Had to move or relocate. | Home life |
|  | 21 | Became homeless. | Home life |
|  | 37 | Unable to get enough food or healthy food. | Economic |
|  | 38 | Unable to access clean water. | Economic |
|  | 39 | Unable to pay important bills like rent or utilities. | Economic |
|  | 40 | Difficulty getting places due to less access to public transportation or concerns about safety. | Economic |
|  | 41 | Unable to get needed medications (e.g., prescriptions or over-the-counter). | Economic |
| Emotional health | 44 | Increase in mental health problems or symptoms (e.g., mood, anxiety, stress). | Emotional health and well-being |
|  | 45 | Increase in sleep problems or poor sleep quality. | Emotional health and well-being |
|  | 47 | Unable to access mental health treatment or therapy. | Emotional health and well-being |
|  | 48 | Not satisfied with changes in mental health treatment or therapy. | Emotional health and well-being |
|  | 49 | Spent more time on screens and devices (e.g., looking at phone, playing video games, watching TV). | Emotional health and well-being |
| Substance use | 46 | Increase in use of alcohol or substances. | Substance use |
|  | 93 | Increased difficulty accessing clean needles | Substance use |
|  | 94 | Increased difficulty accessing naloxone | Substance use |
|  | 95 | Increased difficulty accessing preferred drug of choice | Substance use |
|  | 96 | Increased difficult accessing recovery support services | Substance use |
| Physical health | 50 | Increase in health problems not related to this disease. | Physical health problems |
|  | 51 | Less physical activity or exercise. | Physical health problems |
|  | 52 | Overeating or eating more unhealthy foods (e.g., junk food). | Physical health problems |
|  | 53 | More time sitting down or being sedentary. | Physical health problems |
|  | 54 | Important medical procedure cancelled (e.g., surgery). | Physical health problems |
|  | 55 | Unable to access medical care for a serious condition (e.g., dialysis, chemotherapy). | Physical health problems |
|  | 56 | Got less medical care than usual (e.g., routine or preventive care appointments). | Physical health problems |
| Physical distancing and quarantine | 58 | Isolated or quarantined due to possible exposure to this disease. | Physical distancing and quarantine |
|  | 59 | Isolated or quarantined due to symptoms of this disease. | Physical distancing and quarantine |
|  | 60 | Isolated due to existing health conditions that increase risk of infection or disease. | Physical distancing and quarantine |
|  | 61 | Limited physical closeness with child or loved one due to concerns of infection. | Physical distancing and quarantine |
|  | 62 | Moved out or lived away from family due to a high-risk job (e.g., health care worker, first responder). | Physical distancing and quarantine |
|  | 63 | Close family member not in the home was quarantined. | Physical distancing and quarantine |
|  | 64 | Family member was unable to return home due to quarantine or travel restrictions. | Physical distancing and quarantine |
|  | 65 | Entire household was quarantined for a week or longer. | Physical distancing and quarantine |
|  | 97 | Could not isolate or quarantine due to unstable housing (e.g., living in car, crashing at friends’ houses) | Physical distancing and quarantine |
|  | 98 | Could not isolate or quarantine due to living in close proximity to other individuals outside my family (e.g., recovery housing, shelter) | Physical distancing and quarantine |
| Infection exposure | 66 | Currently have symptoms of this disease but have not been tested. | Infection history |
|  | 67 | Tested and currently have this disease. | Infection history |
|  | 68 | Had symptoms of this disease but never tested. | Infection history |
|  | 69 | Tested positive for this disease but no longer have it. | Infection history |
|  | 70 | Got medical treatment due to severe symptoms of this disease. | Infection history |
|  | 71 | Hospital stay due to this disease. | Infection history |
|  | 73 | Death of close friend or family member from this disease. | Infection history |
| Caretaking | 12 | Had a child in home who could not go to school. | Education and training |
|  | 14 | Childcare or babysitting unavailable when needed. | Home life |
|  | 15 | Difficulty taking care of children in the home. | Home life |
|  | 17 | Had to take over teaching or instructing a child. | Home life |
|  | 19 | Had to spend a lot more time taking care of a family member. | Home life |
|  | 42 | Increase in child behavioral or emotional problems. | Emotional health |
|  | 43 | Increase in child’s sleep difficulties or nightmares. | Emotional health |
| Items excluded from analysis | 4 | Had to continue to work even though in close contact with people who might be infected (e.g., customers, patients, co-workers). | Work and employment |
|  | 5 | Spend a lot of time disinfecting at home due to close contact with people who might be infected at work. | Work and employment |
|  | 9 | Provided direct care to people with the disease (e.g., doctor, nurse, patient care assistant, radiologist). | Work and employment |
|  | 10 | Provided supportive care to people with the disease (e.g., medical support staff, custodial, administration). | Work and employment |
|  | 11 | Provided care to people who died as a result of the disease. | Work and employment |
|  | 13 | Unable to go to school or training for weeks or had to withdraw. | Education and training |
|  | 57 | Elderly or disabled family member not in the home unable to get the help they need. | Physical health problems |
|  | 72 | Someone died of this disease while in our home | Infection history |
|  | 74 | More quality time with family or friends in person or from a distance (e.g., on the phone, Email, social media). | Positive change |
|  | 75 | More quality time with partner or spouse. | Positive change |
|  | 76 | More quality time with children. | Positive change |
|  | 77 | Improved relationships with family or friends. | Positive change |
|  | 78 | New connections made with supportive people. | Positive change |
|  | 79 | Increase in exercise or physical activity. | Positive change |
|  | 80 | More time in nature or being outdoors. | Positive change |
|  | 81 | More time doing enjoyable activities (e.g., reading books, puzzles). | Positive change |
|  | 82 | Developed new hobbies or activities. | Positive change |
|  | 83 | More appreciative of things usually taken for granted. | Positive change |
|  | 84 | Paid more attention to personal health. | Positive change |
|  | 85 | Paid more attention to preventing physical injuries. | Positive change |
|  | 86 | Ate healthier foods. | Positive change |
|  | 87 | Less use of alcohol or substances. | Positive change |
|  | 88 | Spent less time on screens or devices outside of work hours (e.g., looking at phone, playing video games, watching TV). | Positive change |
|  | 89 | Volunteered time to help people in need. | Positive change |
|  | 90 | Donated time or goods to a cause related to this disease (e.g., made masks, donated blood, volunteered). | Positive change |
|  | 91 | Found greater meaning in work, employment, or school. | Positive change |
|  | 92 | More efficient or productive in work, employment, or school. | Positive change |
|  | 99 | Greater access to take home doses. | Positive change |

Additional Table S2

*Results from Inflation Component of Zero-Inflated Binomial Prediction of Follow-Up Opioid-Related Problems (n = 129)*

|  |  | Opioid-Related Problems | | |
| --- | --- | --- | --- | --- |
|  |  |  | 95% CI | |
|  |  | OR | Lower | Upper |
| Model 2 | Baseline | 0.93 | 0.84 | 1.03 |
|  | Employment | 1.03 | 0.74 | 1.43 |
|  | Interpersonal | 0.85 | 0.56 | 1.28 |
|  | Social | 1.04 | 0.84 | 1.30 |
|  | Economical | 0.84 | 0.63 | 1.14 |
|  | Emotional | 1.11 | 0.76 | 1.61 |
|  | Substance Use | 0.68 | 0.41 | 1.13 |
|  | Physical | 1.21 | 0.90 | 1.62 |
|  | Quarantine | 0.96 | 0.71 | 1.32 |
|  | Infection | 0.92 | 0.38 | 2.27 |
|  | Childcare | 1.00 | 0.78 | 1.27 |

*Note*. OR = odds ratio, Model 2 = full model simultaneously controlling for all of the Epidemic-Pandemic Impacts Inventory domains
